# Mass Convention of the Dentists of the West

**Published:** 1872-03

**Authors:** I. Knapp

**Affiliations:** Sec.


### MASS CONVENTION OF THE DENTISTS OF THE WEST.

The called Mass Convention of the Dentists of the West, was held in the Dental College of Ohio, on College street, Cincinnati, March 7th, 1872, and was organized by calling Dr. W. H. Morgan, of Nashville, to the Chair, and appointing Dr. I. Knapp, of Fort Wayne, Indiana, Secretary. Dr. Rehwinkle, of Chillicothe, Ohio, stated the object of the Convention to be the consideration of the case of the Goodyear Dental Vulcanite Company against Benoni E. Gardner, which has been selected by the profession for a test case. The point of Dr. Rehwinkle's remarks was that if the profession are successful in this suit, the patent will be declared null and void, and thus relieve the Dentists throughout the country from the exactions of the company.

Dr. W. P. Horton, of Cleveland, Ohio, gave a succinct history of the organization and practices of the company, and the litigation which has resulted from their attempts to exact tribute from the profession, and concluded by saying that he was authorized by the Dentists in his section to say that they are willing to bear their share of expenses incurred in the defense of the Gardner Case.

The subject was discussed at some length by Drs. Morgan, Rehwinkle, Smith and Horton. After which Dr. Rosson moved that a committee of three be appointed to consider and present a plan of action. Motion lost.

Dr. Taylor offered the following which were adopted:

*Resolved*, That this Convention does not deem it expedient or desirable to employ at present, additional counsel in the Gardner Case.

*Resolved*, That this convention take immediate measures to raise funds to aid in defraying the expenses of the Gardner Case.

On motion, the following named gentlemen were appointed as a Finance Committee, to solicit aid from the profession:

Dr. James Taylor, Cincinnati; Dr. E. Osmond, Cincinnati; Dr. Isaac Knapp, Fort Wayne, Indiana; Dr. Wm. N. Morrison, St. Louis; Dr. P. G. C. Hunt, Indianapolis, Indiana.

On motion, James Leslie, Esq., of Cincinnati, was appointed Treasurer of the fund to be raised as provided.

Dr. H. A. Smith, of Cincinnati, presented the following, which was adopted:

*Resolved*, That the interest which Dr. S. S. White has taken in the litigation now pending, involving the validity of the Cummings patent—by giving both his time and means—deserves the gratitude of the whole dental profession; and that we tender Dr. White a vote of thanks:

Dr. Rosson presented the following, which was adopted:

*Resolved*, That this Convention indorse the committee of the Ohio Dental Society in the action it has taken to relieve the profession of the burden of the rubber patents, and pledge them all needed moral aid and pecuniary support in any future action to secure the end desired.

The Convention then adjourned sine die.

I. KNAPP, *Sec.*

